# Factors associated with parental smoking in the presence of school-aged children: a cross-sectional study

**DOI:** 10.1186/1471-2458-13-819

**Published:** 2013-09-10

**Authors:** Yuan-Mei Liao, Yu-Ting Chen, Liang-Chun Kuo, Ping-Ling Chen

**Affiliations:** 1 Graduate Institute of Nursing, College of Nursing, Taipei Medical University, No. 250, Wu-Hsing Street, Taipei City 110, Taiwan; 2 School of Nursing, College of Medicine, Chang-Gung University, No. 259, Wen-Hwa 1st Road, Kwei-Shan, Tao-Yuan 333, Taiwan; 3 Graduate Institute of Injury Prevention and Control, College of Public Health and Nutrition, Taipei Medical University, No. 250, Wu-Hsing Street, Taipei City 110, Taiwan

**Keywords:** Parental smoking, Environmental tobacco smoke, Children, Perception, Family influence

## Abstract

**Background:**

In 2009, the Tobacco Hazards Prevention Act (Taiwan) was amended to more effectively restrict smoking in indoor public places and workplaces in Taiwan. However, the lack of prohibitions for smoking in private homes may place family members at increased risk for exposure to environmental tobacco smoke (ETS). The aim of our study was to determine the factors associated with parental smoking in the presence of children at home.

**Methods:**

In 2010, we performed a cross-sectional study of factors associated with parental smoking in the presence of children at home in Taiwan using self-administered questionnaires. Quota sampling was used to select five primary schools from four different regions of Taiwan. Parents were surveyed to identify parental smokers and 307 parental smokers were selected for participation in our study. Questionnaire data regarding parental smoking in the presence of children at home and related interactions among family members were analyzed. Hierarchical logistic regression was used to determine the best-fit model for examining the relationships among the variables related to parental smoking in the presence of children at home.

**Results:**

Two-thirds of parents who smoked reported smoking in the presence of their children. The results of the hierarchical logistic regression analysis identified the smokers’ compliance with their family’s antismoking responses, mutual agreement with smoking bans, daily smoking, smoking more than 20 cigarettes per day, the education level of the parental smoker, and the annual family income as determinants of smoking in the presence of children at home.

**Conclusions:**

Households with smoking parents should be targeted for interventions to encourage the adoption and enforcement of home smoking bans. Educational interventions that promote smoke-free homes for children and provide support to help parents stop smoking are critical factors in reducing the frequency of children’s ETS exposure in the home.

## Background

Antismoking policies, rising tobacco taxes, increased health welfare surcharges, the restriction of tobacco advertisements, health warnings on tobacco packaging, and smoking-cessation campaigns have contributed to a gradual decline in the prevalence of smoking in Taiwan [1]. However, the rate of smoking among men in Taiwan has remained high, with a prevalence of 38.6% for men aged over 18 years in 2008 [1]. The Tobacco Hazards Prevention Act (THPA) restricts smoking indoors in public places and public transportation facilities in Taiwan. The THPA was amended in 2009 to include indoor workplaces with three or more people. Violators of the THPA can be fined from NT$10,000 to $50,000.

Assessments of the impact of smoke-free legislation on children’s exposure to environmental tobacco smoke (ETS) at home have varied. A previous study in the United States showed that substantial restrictions of smoking in public places and workplaces resulted in smokers’ homes becoming the predominant location for smoking [2]. Two studies from England and Wales reported no change in children’s exposure to ETS at home after the implementation of smoke-free legislation [3,4]. Although investigators have concluded that banning smoking in public places has no adverse influence on children’s exposure to ETS at home, the frequency of children’s exposure to ETS was reported to be highest in their homes [3]. Another study in Hong Kong reported an increase in children’s exposure to ETS at home following the implementation of comprehensive smoke-free legislation [5]. In Taiwan, approximately 41% to 50% of junior/senior high school students are exposed to ETS at home, and about half of them exposed to ETS daily [1,6]. The architecture of high-rise urban dwellings in Taiwan is similar to that of Hong Kong; both limit residential living spaces. Smoke-free legislation that excludes private homes may contribute to a relatively high prevalence of ETS exposure at home [7].

Exposure at home has been identified as the most common type of children’s exposure to ETS [2]. Children living with smokers are at 1.40 times greater risk (95% CI = 1.23-1.60) for emotional or behavioral problems than children living with nonsmokers, and the risk increases with increasing numbers of smokers in the household [8]. Compared with adults, children have a greater risk of adverse health-related effects from chronic exposure to ETS because they breathe faster and have smaller, less-developed lungs [9]. The threats to children’s health posed by ETS exposure include adverse physical effects, such as lung function abnormalities, asthma, ear disorders, and sudden infant death syndrome [2,10], as well as behavioral, attitudinal, and emotional dysfunctions [8,11].

Parents who know of the harmful effects associated with ETS exposure demonstrate more effective strategies to prevent their children’s exposure to secondhand smoke [12,13]. However, increasing one’s knowledge of the harmful effects of ETS exposure may not be effective in promoting quitting [14] or restricting smoking at home [15]. Certain parents perceive ETS risk based on their personal experiences [16], whereas others underestimate the risk of ETS exposure [17]. Parents’ perceptions of parental smoking and their views of its impact on children influence their decisions to smoke in the presence of their children [18]. Qualitative studies have shown that parental smokers who were made aware of their family’s objections to smoking were more likely to decide not to smoke around their children [18] or to restrict their smoking at home [15], whereas a failure to openly discuss restrictions of household smoking was perceived by smokers to represent their family’s acceptance of smoking at home [17]. Data from quantitative surveys of the influence of families’ responses on parental smoking are scant, but previous studies have shown that, despite having perceptions of the dangers of ETS exposure, home smoking bans were uncommon among parental smokers, particularly among those with older children [19,20]. Less than 20% of parental smokers were consistently compliant with restrictions on smoking in the home [21,22].

Previous studies of the relationships between demographic factors and smoking showed that parents’ age, gender, socioeconomic status, and smoking experience correlated with both the knowledge of ETS-related health threats [23] and children’s exposure to ETS [12,24]. Thus, we proposed that such demographic characteristics may also be associated with parental smoking in the presence of children at home. In our study, we examined the factors associated with parental smoking in the presence of school-aged children, including parental smokers’ awareness of the risks of ETS exposure, their family’s responses to smoking at home and its influence on parental smoking, the implementation of home smoking bans, and the parents’ demographic characteristics.

## Methods

### Study design

A cross-sectional study was conducted using self-administered questionnaires to collect data from parental smokers in 2010. Our study was approved by the Institutional Review Board of Taipei Medical University (IRB No. P960233). For participant selection, we used quota sampling to divide the counties and cities of Taiwan into four regions, northern, central, southern, and eastern, according to their level of urbanization and access to resources. We selected five primary schools, one each from the middle, southern, and eastern regions, and two from the northern region. Two classes were selected at random from Grades 1 through 6 at each school. To avoid gender bias in our selection of parental smokers, each set of paired classes was assigned randomly to a cluster to invite either the fathers or the mothers of the students to participate in our study.

An invitation letter describing the study, a consent form, and a self-administered anonymous questionnaire were sent to the parents, who voluntarily completed and returned the consent form and questionnaire to the school in a sealed envelope. To ensure that only current smokers were included in our analysis, parental smokers were defined as those parents who reported smoking more than 100 cigarettes in their lifetime and smoking on more than one day during the preceding month. The variable, “smoking in the presence of children,” was defined as parental smokers smoking in the presence of their children in the same room at home. Parental smokers were categorized into one of the two parental-smoker study groups based on whether they answered “yes” or “no” to the questionnaire item, “Do you smoke in the presence of your children when you and your children are in the same room?” The smoking-related and demographic variables were examined to identify possible associations with the different parental-smoker groups.

### Measures

#### Demographic variables

The parental smokers’ gender, age (21–40 years or 41–60 years), marital status (married or unmarried), and family type (nuclear or mixed) were treated as dichotomous variables (Table 1). The parental smokers were classified into one of three categories based on their level of education, their occupation status, and the grade levels of their children. The parental smokers’ households were classified as earning an annual income of less than NT$600,000 or NT$600,000 and over (approximately US$20,000).

**Table 1:** Associations between the demographic characteristics of parental smokers and smoking in the presence of their children

**Variables**	**Smoked in the presence of children**		**Did not smoke in the presence of children**		**OR**	**95% CI**
	***n***	**%**	***n***	**%**		
Overall (*N* = 307)	203	66.12	104	33.88	-	
Gender						
Male	171	64.29	85	35.71		
Female	32	80.00	8	20.00	2.22	0.98-5.01
Age (y)						
41-60	97	61.78	60	38.22		
21-40	104	70.27	44	29.73	1.46	0.91-2.36
Marital status						
Married	161	65.45	85	34.55		
Unmarried	38	69.09	17	30.91	1.18	0.63-2.21
Education						
≥Baccalaureate	54	55.10	44	44.90		
High School	111	70.70	46	29.30	1.97	1.16-3.33^*^
Junior high	36	75.00	12	25.00	2.44	1.14-5.25^*^
Occupation						
Labor	77	65.25	41	34.75		
Business/Service	96	62.75	57	37.25	0.90	0.54-1.48
Other	27	84.38	5	15.63	2.88	1.03-8.03
Family type						
Nuclear	104	66.67	52	33.33		
Mixed	98	65.33	52	34.67	0.94	0.59-1.51
Annual income						
>$600,000	65	58.04	47	41.96		
≤$600,000	131	71.58	52	28.42	1.82	1.11-2.99^*^
Grades of children						
1-2	62	63.27	36	36.73		
3-4	74	70.48	31	29.52	1.38	0.77-2.49
5-6	67	64.42	37	35.58	1.05	0.59-1.87
Region of Taiwan						
North	67	62.04	41	37.96		
Central	39	65.00	21	35.00	1.13	0.59-2.19
South	52	70.27	22	29.73	1.45	0.77-2.72
East	45	69.23	20	30.77	1.38	0.72-2.65

OR, odds ratio; CI, confidence interval.

*p < .05.

#### Tobacco use-related variables

Three variables were used to assess the smoking habits of parents (Table 2). Daily smoking (yes or no) and the number of cigarettes smoked per day (≤ 20 or ≥ 21) were treated as dichotomous variables. Parental smokers were categorized based on the age at which they smoked their first cigarette (>18 years or ≤ 18 years). An attempt to quit smoking during the year preceding the study period was treated as a dichotomous variable (attempted or did not attempt). Thoughts of quitting smoking and receiving assistance from health-care professionals to stop smoking were categorized into one of three levels, as shown in Table 2.

**Table 2:** Associations between smoking in the presence of children at home and variables related to smoking habits, quitting smoking, and smoking bans in the home

**Variables**	**Smoked in the presence of children**		**Did not smoke in the presence of children**		**OR**	**95% CI**
	***n***	**%**	***n***	**%**		
Daily smoker						
No	30	50.85	29	49.15		
Yes	173	69.76	75	30.24	2.23	1.25-3.97^*^
Cigarettes smoked per day (n)						
<20	109	58.60	77	41.40		
≥20	91	78.45	25	21.55	2.57	1.51-4.37^*^
Age at first cigarette smoked (y)						
>18	62	59.62	42	40.38		
≤18	139	69.85	60	30.15	1.57	0.96-2.58
Ever considered quitting						
Yes, in coming year	40	56.34	31	43.66		
Yes, but not in coming year	91	65.00	49	35.00	1.43	0.80-2.58
No	66	74.16	23	25.84	2.22	1.14-4.33^*^
Attempted to quit in preceding year						
Yes	110	62.15	67	37.85		
No	93	71.54	37	28.46	1.53	0.94-2.49
Advised to quit by health-care professional						
Yes	77	65.25	41	34.75		
No	70	69.31	31	30.69	1.20	0.68-2.12
No contact with health- care professionals	55	63.95	31	36.05	0.95	0.53-1.69
Agreed with home smoking bans						
Yes	140	61.67	87	38.33		
No	59	77.63	17	22.37	2.16	1.18-3.94^*^
Had smoking bans at home						
Yes	51	49.04	53	50.96		
No	149	76.41	46	23.59	3.37	2.03-5.59^*^
Enforcement of smoking bans at home						
Consistent	21	39.62	32	60.38		
Inconsistent	34	68.00	16	32.00	3.24	1.44-7.28^*^

OR, odds ratio; CI, confidence interval.

*p < .05.

#### Smoking bans at home

The questionnaire item, “Do you have smoking bans at home?” was used to identify homes with current smoking bans. The questionnaire item, “Do you agree with smoking bans at home?” was used to assess parental smokers’ attitudes regarding the antismoking responses of their families. The questionnaire item, “Does your family consistently enforce smoking bans at home?” was used to assess compliance with home smoking bans. The responses to these questions were treated as dichotomous variables (Table 2).

#### Parental perceptions, consequence evaluations, families’ antismoking responses, and smokers’ reactions to families’ antismoking responses

Four scales were developed based on the findings of a preliminary qualitative study [18], and were used to assess parental smokers’ perceptions, consequence evaluations, their family’s antismoking responses, and smokers’ reactions to their family’s request not to smoke in the presence of children at home. We examined the psychometric properties of the scales by performing exploratory factor analysis, and internal-consistency and known-group validity analyses of the questionnaire data. The results of factor analysis revealed two to three factors within each scale. Cronbach’s alpha was between .63 and .91 for the scales and subscales, respectively. The results of known-group validity testing demonstrated an ability to discriminate between groups of parents who smoked and those who did not smoke in the presence of their children at home.

Nine items comprised the first scale, which was used for assessing the parental smokers’ perceptions of smoking in the presence of their children (PSPC) by ascertaining parents’ beliefs regarding PSPC. Six items comprised the second scale, which was used to assess the parental smokers’ evaluations of the consequences of smoking in the presence of their children (CESPC) by examining the parents’ judgments of the outcomes of such acts. Fifteen items comprised the third scale, which was used for assessing the antismoking responses of the family members of parents who smoked in the presence of their children (FRSPC). Twenty-two items comprised the fourth scale, which was used to assess the parental smokers’ reactions to their respective family’s antismoking responses (RTFR). Responses were scored based on a 5-point Likert scale, with 1 indicating completely disagree and 5 indicating completely agree.

Higher scores on the PSPC and CESPC scales indicated more negative perceptions and more accurate consequence evaluations. A higher score on the RFSPC scale indicated that parental smokers perceived a greater number of antismoking responses from other family members. Parental smokers who were more compliant with the antismoking responses of their family scored higher on the RTFR scale than those who were less compliant. The Cronbach’s alpha was .79, .81, .87, and .90 for PSPC, CESPC, FRSPC, and RTFR, respectively.

### Data analysis

Bivariate associations between smoking or not smoking in the presence of children at home and the demographic variables, smoking frequency and history, quitting attempts, and the existence of smoking bans at home were assessed using a chi-squared analysis. Logistic regression was used to estimate the likelihood of whether smoking in the presence of children at home was associated with attitudes, outcome evaluation, the subjective norms expressed by family members, and the smokers’ responses to the subjective norms. Significant bivariate associations were included in the hierarchical logistic regression models, and the fitness of the models was assessed based on a difference of −2 times the log likelihood of the data (−2LL). All analyses were conducted using SAS computer software (version 9.1; SAS Institute, Cary, NC, USA).

## Results

### Demographic characteristics and smoking in the presence of children

Among the 1,670 selected parents, 1,436 returned questionnaires; a response rate of 86%. Of those parents, 46% (n = 661) were fathers and 54% (n = 775) mothers. Among the responding parents, 43% of fathers (281 in 661) and 6% of mothers (47 in 775) were current smokers. About 93.6% of parental smokers (307 in 328) completed questionnaires to a sufficient degree and were included in data analysis. Among these 307 currently smoking parents, 66% reported smoking in the presence of their children. The probability of smoking in the presence of children was 2.44 times higher (95% confidence interval [CI] = 1.14–5.25) for parental smokers with a junior high school level education or below than for those with a baccalaureate or above. Compared with parental smokers with a baccalaureate or above, parental smokers with a high school education smoked 1.97 times more frequently (95% CI = 1.16–3.33) in the presence of their children. Parental smokers living in households with an annual income of less than NT$600,000 smoked in the presence of their children 1.82 times more frequently (95% CI = 1.11–2.99) than did parental smokers living in households with an annual income of NT$600,000 or more. Smoking mothers reported smoking in the presence of their children more frequently than smoking fathers, but the difference reached borderline statistical significance only (80% vs. 64%; *p* = .05). Other demographic characteristics, such as age, marital status, occupation, family type, children’s grade levels, and the region of residence, were not associated with smoking or not smoking in the presence of children (Table 1).

### Smoking habits

Frequent smoking was associated with smoking in the presence of children (Table 2). Daily smokers were 2.23 times (95% CI = 1.25–3.97) more likely to smoke in the presence of their children than were parents who were not daily smokers. Parents who smoked 20 cigarettes or more per day were 2.57 times (95% CI = 1.51–4.37) more likely to smoke in the presence of their children than were parents who smoked less frequently. Compared with smokers who were considering quitting smoking in the coming year, smokers who were not considering quitting were more likely to smoke in the presence of their children (OR = 2.22, 95% CI = 1.14–4.33). In the variable, smoking in the presence of their children, no statistically significant differences were observed among parents who first smoked at age 18 years or younger and those who smoked their first cigarette later in life, parental smokers who received advice from a health professional regarding quitting and those who had not, or parental smokers who had attempted to quit in the preceding year and those who had not (Table 2).

### Smoking bans at home

The establishment and enforcement of smoking bans in the home were associated with less frequent smoking around children (Table 2). Parental smokers who disagreed with smoking bans at home were 2.16 times (95% CI = 1.18–3.94) more likely to smoke in the presence of children at home than were parental smokers who agreed with smoking bans. Parental smokers who lived in homes that lacked smoking bans were 3.37 times (95% CI = 2.03–5.59) more likely to smoke in the presence of children than were those who had smoking bans at home. Parental smokers who lived in homes with smoking bans that were not consistently enforced were 3.24 times (95% CI = 1.44–7.28) more likely to smoke in the presence of their children than were parental smokers who lived in homes in which smoking bans were consistently enforced.

### Parental perceptions, consequence evaluation, family’s antismoking responses, and smokers’ reactions to family’s antismoking responses

Parental smokers’ perceptions of smoking in the presence of children, their evaluations of the consequences of smoking around children, their family’s antismoking responses to smoking in the home, and their reactions to their family’s antismoking responses were associated with the probability of parents smoking in the presence of their children (Table 3). An increase in a parents’ PSPC score of 1 was associated with a 0.93-fold decrease (95% CI = 0.89–0.97) in the likelihood of parents smoking in the presence of their children. The acknowledgment of a greater number of negative consequences of smoking in the presence of children was associated with a lower probability of parents smoking in the presence of their children (OR = 0.88, 95% CI = 0.82–0.93). Parental smokers who perceived more antismoking responses from other family members (OR = 0.95, 95% CI = 0.93–0.97), as well as those who were more compliant with their family’s antismoking responses (OR = 0.92, 95% CI = 0.90–0.95) were also less likely to smoke in the presence of their children.

**Table 3:** Associations between smoking in the presence of children at home and parental smokers’ perceptions and evaluations of the consequences of children’s exposure to second-hand smoke, their families’ anti-smoking responses, and the smokers’ reactions to the anti-smoking responses

**Variables**	**Total score**		**OR**	**95% CI**
	**Mean**	**SE**		
Perceptions of smoking in the presence of their children (PSPC)	32.60	0.33	0.93	0.89-0.97^*^
Evaluations of the consequences of smoking in the presence of children (CESPC)	23.03	0.23	0.88	0.82-0.93^*^
Family’s anti-smoking responses to parental smoking in the presence of children (FRSPC)	43.07	0.64	0.95	0.93-0.97^*^
Smokers’ reaction to family’s anti-smoking responses (RTFR)	69.95	0.69	0.92	0.90-0.95^*^

OR, odds ratio; CI, confidence interval.

*p < .05.

### Determinants of smoking behavior in the presence of children at home

The results of the hierarchical logistic regression analysis are shown in Table 4. The first model tested the contribution of RTFR to smoking around children and found statistical significance (−2LL = 342.39, p < 0.05). Adding FRSPC, ECSPC, and PSPC did not improve the fitness of Model 2 compared with Model 1. The addition of the *agreed with smoking bans in the home* and *had smoking bans in the home* data sets in Model 3 resulted in a significant increase in model fitness (−2LL = 313.64, likelihood ratio [LR] χ^2^ = 22.57, p < 0.05). The inclusion of smoking frequency and intensity variables significantly increased the fitness of Model 4 (−2LL = 305.51, LR χ^2^ = 8.13, p < 0.05).

**Table 4:** Results of hierarchical logistic regression modeling to predict smoking in the presence of children at home

	**Model 1**		**Model 2**		**Model 3**		**Model 4**		**Model 5**		**Model 6**	
**Predictors**	**OR**	**95% CI**	**OR**	**95% CI**	**OR**	**95% CI**	**OR**	**95% CI**	**OR**	**95% CI**	**OR**	**95% CI**
RTFR	0.92	0.90-0.95	0.93	0.91-0.96	0.93	0.90-0.95	0.93	0.91-0.96	0.94	0.91-0.96	0.94	0.91-0.96
FRSPC			0.98	0.96-1.01								
CESPC			0.94	0.87-1.01								
PSPC			1.02	0.97-1.08								
Agreed with smoking bans at home (ref: yes)					1.61	0.59-2.30	1.25	0.62-2.53	1.28	0.62-2.62	1.44	0.69-2.98
Had smoking bans at home (ref: yes)					2.57	1.48-4.46	2.70	1.54-4.74	2.58	1.46-4.57	2.78	1.55-4.99
Daily smoker (ref: no)							1.59	0.80-3.17	1.56	0.76-3.20	1.47	0.72-3.01
Smoked ≥ 20 cigarettes per day (ref: ≤ 19)							1.58	0.84-2.96	1.55	0.82-2.93	1.76	0.89-3.47
Ever considered quitting (ref: yes, in the coming year)												
Yes, but not in coming year									1.15	0.58-2.25		
No									0.99	0.42-2.33		
Quitting attempts in the preceding year (ref: no)									0.96	0.51-1.80		
Education level (ref: ≥ Baccalaureate)												
High School											1.11	0.58-2.13
Junior High School and below											0.86	0.31-2.38
Annual income (ref: ≥ $600,001)												
<$600,000											1.71	0.92-3.17
−2LL	342.39		336.21	χ^2^	313.64		305.51	χ^2^	302.02	χ^2^	288.69	χ^2^
Likelihood ratio (model no. vs. model no.)			2 vs. 1	6.18	3 vs. 1	22.57^*^	4 vs. 3	8.13^*^	5 vs. 4	3.49	6 vs. 4	16.82^*^

PSPC, perceptions of smoking in the presence of their children; CESPC, evaluations of the consequences of smoking in the presence of children; FRSPC, family’s anti-smoking responses to parental smoking in the presence of children; RTFR, smokers’ reaction to family’s anti-smoking responses; OR, odds ratio; CI, confidence interval; -2LL, -2 times the log likelihood of the data.

^*^p < .05.

Although Model 5 did not demonstrate improved fitness, the addition of the parent’s level of education and annual income to Model 5 improved the fitness of Model 6 compared with that of Model 4 (−2LL = 288.69, LR χ^2^ = 16.82, p < 0.05). The inclusion of the RTFR, agreement to home smoking bans, had home smoking bans, smoking frequency and intensity, parent smoker’s level of education, and annual income variables in the final model resulted in the best model fitness for predicting parental smoking in the presence of their children. In the final model, parental smokers who were more compliant with their family’s antismoking responses (OR = 0.94, 95% CI = 0.91–0.96) and who had smoking bans in their homes (OR = 2.78, 95% CI = 1.55–4.99) were independ-ently associated with reduced smoking in the presence of their children.

## Discussion

Our results show that parental smokers who considered smoking in the presence of children at home to be acceptable or acknowledged fewer potential negative effects of their children’s exposure to ETS were more likely to smoke in the presence of their children. Previous studies have investigated the relationships between the knowledge of the harmful effects of ETS and children’s exposure to ETS. However, smokers’ perceptions of smoking and their need to smoke might also affect their smoking behavior at home [15,16,18]. Therefore, greater focus must be placed on smokers’ perceptions of the risks of ETS exposure in studies of factors that contribute to smoking in the home [12]. The authors of a systematic review [14] suggested that emphasis should be placed on the risks of children’s ETS exposure based on their findings that emphasizing the protection of children from ETS exposure was associated with an increased cessation rate among parental smokers.

Our study focused on parental smokers’ perceptions of smoking around children and their evaluations of the consequences of children’s ETS exposure, rather than on their knowledge regarding the health-related risks of ETS exposure. Parents who smoked around their children acknowledged fewer negative effects of secondhand smoke on their children and their home environment (air quality, smell). Farber et al. [25] suggested that parents who expose their children to ETS often underestimate the potential harmful effects on their children’s health. Parental smokers must be made aware that there is no risk-free level of exposure to ETS [2].

Jones et al. [15] observed that parental smokers’ need to smoke for stress relief and desire to smoke in a private, comfortable setting were barriers to restricting smoking at home. Jones et al. suggested providing tailored information that considers barriers and motivators for maintaining smoking restrictions in the home. A pattern of reasoning among parental smokers has been identified, in which their beliefs regarding smoking around children, their views of its impact on children, and their personal need to smoke are the primary determinants of parental smoking in the presence of their children [18]. Our study revealed a higher level of acceptance of smoking around children among parents who smoked. According to the subconcepts within the PSPC scale (viewing parental smoking as *adverse behavior*, *harmless behavior*, and *rational needs*) [26], a higher level of acceptance of parental smoking in our study also reflects a more concerns that the effects of parental smoking are justified by their need to smoke. These observations imply that, in addition to emphasizing the negative effects of children’s ETS ex-posure, suggesting healthy substitutes for smoking may also diminish the frequency of parental smoking in the presence of their children.

In our study, the families’ antismoking responses to parental smokers and parental smokers’ reactions to these familial responses were also associated with reduced smoking in the presence of children at home. Parental smokers who perceived more antismoking responses from other family members or were in agreement with and complied more frequently with their family’s antismoking responses smoked less around their children. A previous study determined that a family’s inability to restrict smoking in the home might be because of the acceptance of exposure to ETS as a social interaction and cultural issue [17]. However, other studies have shown that the family’s influence is a motivating factor in maintaining a smoke-free home [15]. Thus, promoting social norms that reduce household smoking is suggested. Family members’ antismoking attitudes are associated with smokers’ intentions to quit and their abstinence from smoking [27]. Our findings support the role of family influence on parental smoking, and the involvement of entire families in prevention programs aimed at reducing children’s ETS exposure.

Our study showed that home smoking bans and the consistent enforcement of such bans also significantly reduced parental smoking in the presence of their children. Previous studies have shown that smoking bans are associated with a reduction in children’s ETS exposure [28]. The amendment to the THPA in 2009 enforced greater restrictions of smoking in public places only. To prevent the possibility of moving smoking into the home, the promotion of bans on smoking at home is critical in preventing children’s exposure to ETS from parental smoking. In addition, health-care providers must improve their efforts to assist entire families in initiating smoking bans in the home.

The efforts of family members and health-care providers notwithstanding, addiction to tobacco use remains the major barrier to restricting home smoking, and must be addressed by any effort to maintain a ban on smoking in the home [12]. Our study showed that daily smoking and smoking more than 20 cigarettes per day had an additive effect on the frequency of smoking in the presence of children at home. These findings are consistent with those of previous studies, which determined that daily smoking and higher numbers of cigarettes smoked per day were strong predictors of parental smoking in the home [21,24]. Heavy smoking may reflect a higher level of nicotine dependence, and it may be more difficult for heavy smokers to refrain from smoking under any set of circumstances, compared with smokers who smoke less frequently. Therefore, it is likely that heavy smokers will restrict their smoking in the presence of children at home less frequently. The effectiveness of programs designed to prevent children’s exposure to ETS may benefit from the inclusion of nicotine-replacement therapy, such as the use of nicotine patches, for parental smokers [14]. However, one study reported that the use of nicotine-replacement therapy did not result in reduced exposure of children to ETS in the home [29]. Thus, a more detailed understanding of the effects of nicotine-replacement therapy on smokers is needed to support the inclusion of nicotine-replacement therapy in ETS-prevention programs.

Parental smokers who were considering quitting smoking during the coming year smoked less around their children. This finding implies a relationship between a reduced frequency of smoking in the presence of children at home and the parental smokers’ intentions to quit. In addition, previous studies have shown that parents are more willing to accept help in quitting smoking when health-care providers provide information regarding the benefits of a smoke-free home, rather than information regarding methods of quitting smoking [30]. Parents perceive that making their home smoke-free may help them to quit in the long run [15]. Thus, promoting a smoke-free home environment for children might encourage parental smokers to quit smoking.

Our study shows that demographic predictors of parental smoking indicate that specific groups may need to be targeted for intervention to reduce children’s exposure to ETS effectively. Parents who were less educated and lived in households with lower annual incomes smoked more frequently in the presence of their children. These findings are consistent with those of previous studies [12,23], which suggested that providing information tailored specifically for these demographic groups may be more effective in reducing smoking in the home. These groups of parental smokers should also be targeted for improved health education regarding the harmful effects of children’s ETS exposure and strategies to promote a smoke-free home environment. A series of studies in Scandinavian countries demonstrated that long-term nationwide campaigns aimed at parental smokers may substantially reduce children’s ETS exposure [31-33]. National public-health systems must therefore establish effective approaches to address the prevention of children’s ETS exposure resulting from parental smoking specifically. We suggest that schools may also be a useful resource to support ETS-prevention programs for children through relationships between parents and schoolteachers or administrative staff.

The participants in our study were selected from four representative locations throughout Taiwan (northern, central, southern, and eastern), which lends credibility to our findings. However, the relatively small sample of parental smokers surveyed does limit the generalization of our findings. More predictors might have been identified if more participants had been included. The lack of an assessment of parents’ nicotine dependency and numbers of parents smoking at home is also a potential limitation of our findings, since these factors may also influence parents’ decisions to smoke in the presence of their children. The use of convenience sampling and the cross-sectional design of our study may also reduce inferences of possible causal relationships among the factors that contribute to smoking around children. Therefore, the use of random sampling and a longitudinal study design are suggested for future studies of parental smoking in the presence of school-aged children.

## Conclusions

Parental smoking in the presence of children at home is associated with their family’s antismoking responses and the adoption of home smoking bans. Parents who smoke around their children demonstrate a greater acceptance of children’s ETS exposure and acknowledge fewer negative effects of ETS exposure on children’s health. This study is the first in Taiwan to target parental smokers of school-aged children by surveying their actions, perceptions, and their family’s influence on their decisions to smoke in the presence of their children. Our findings can be applied to ETS-prevention programs, including educational interventions and the promotion of smoke-free homes for children accompanied by smoking-cessation support for parents. The involvement of both parental smokers and their families in ETS-prevention programs is suggested to ensure the maintenance of home smoking bans.

## Competing interests

The authors declare no competing interests.

## Authors’ contributions

All authors contributed to the design of the study. YML was responsible for literature searches, the interpretation of the results, and writing the manuscript. YTC and LCK participated in the development of the study protocol, data collection, and analysis, and contributed to the drafting of the manuscript. PLC was responsible for the conception of the study and overall supervision of the data collection and analysis, the interpretation of the results, and manuscript preparation. All authors have read and approved of the final manuscript.

## Pre-publication history

The pre-publication history for this paper can be accessed here:

http://www.biomedcentral.com/1471-2458/13/819/prepub
